# Using Sound to Simulate Tactile Cues: AI-Generated Audio and Pseudo-Haptics in Medical Simulation

**DOI:** 10.7759/cureus.108297

**Published:** 2026-05-05

**Authors:** Sandy Abdo, Bill Kapralos, KC Collins, Adam Dubrowski

**Affiliations:** 1 Health Sciences, Faculty of Science, Ontario Tech University, Oshawa, CAN; 2 Medical Education and Simulation, maxSIMhealth Group, Ontario Tech University, Oshawa, CAN; 3 Interactive Multimedia and Design Program, School of Information Technology, Carleton University, Ottawa, CAN; 4 Medical Education and Simulation, Faculty of Health Sciences, Ontario Tech University, Oshawa, CAN

**Keywords:** ai-generated audio, generative ai, immersive virtual learning environment (ivle), pseudo-haptics, psychomotor skill development

## Abstract

Immersive virtual learning environments (iVLEs) are increasingly used in medical, yet psychomotor skill development within these platforms remains constrained by limited access to high-fidelity haptic technologies. Pseudo-haptics, which uses non-tactile sensory cues to influence tactile perception, offers an alternative, though existing work has focused largely on visual modalities. This editorial examines the role of auditory feedback in procedural medicine and explores AI-generated audio as a complementary pseudo-haptic channel in iVLEs for medical training. Drawing on drilling-based procedures such as intraosseous access, we discuss how audio-tactile interactions influence procedural control and safety. An experimental approach is proposed to evaluate how adaptive AI-generated audio could reinforce tactile perception in iVLEs without reliance on physical haptic hardware. AI-generated audio may provide a scalable and accessible pseudo-haptic strategy for enhancing sensory realism and supporting psychomotor training in iVLEs for medical training.

## Editorial

Immersive virtual learning environments (iVLEs) are increasingly being applied to medical education for teaching clinical knowledge and decision-making. Evidence suggests that iVLEs are effective for developing cognitive and affective competencies, particularly when compared to traditional didactic approaches [1]. However, their application to psychomotor skill acquisition remains limited. Tactile cues are a vital component in psychomotor skill development, yet simulating tactile cues is both difficult and costly, requiring high-fidelity haptic devices often not available at the consumer level, thus restricting their use to specialized simulation centers [2]. Throughout this editorial, tactile cues refer to perceptual signals associated with touch, whereas haptic feedback refers specifically to the physical delivery of such cues via hardware-based systems [3].

However, emerging research in immersive learning and human-computer interaction suggests that realism in simulation is not solely a material property but a perceptual phenomenon shaped by multimodal sensory integration and embodied experience [4,5]. From this perspective, simulation environments function not only as physical systems but also as interactive perceptual systems whereby sensory cues jointly construct meaning.

A potential approach to addressing the limitations of high-fidelity haptic devices is the use of pseudo-haptics, that is, the perception of tactile cues through non-tactile sensory cues [3,6]. Pseudo-haptics research has predominantly focused on visual manipulation [3,6,7], with little attention paid to the role of auditory cues in forming tactile perception. This gap is notable, given that sound plays a critical role in many real-world medical procedures. Recent advances in AI-generated audio provide an opportunity to revisit this imbalance and explore audio as an integral component of pseudo-haptics within iVLEs. This editorial aims to examine the role of AI-generated auditory cues as a scalable pseudo-haptic channel in iVLEs and to explore their potential to enhance psychomotor skill development in medical simulation.

The role of auditory cues in procedural medicine

In numerous medical procedures, tactile perception cannot be separated from auditory information. For example, dental osteotomy, joint replacement surgery, craniectomy, and intraosseous (IO) infusion all rely on changes in pitch, intensity, and vibration to signal transitions in material density and resistance [8,9]. In IO infusion, an emergency procedure where a needle is inserted into the bone marrow to quickly deliver fluids, medications, or nutrients when intravenous access is difficult [8], precision drilling in bones is needed to avoid damaging blood vessels and nerves. Practitioners rely on auditory and tactile cues to assess depth, as visual cues are limited. When the drill reaches the spongier bone marrow, they experience a reduction in resistance (tactile cues) and perceive changes in pitch and intensity (auditory feedback), signaling them to stop, ensuring accuracy and safety [9,10]. From an embodied cognition perspective, such auditory cues are not supplementary but constitutive of procedural expertise, emerging through the coupling of perception and action during skilled performance [4].

Despite this reliance on sound in real-world practice, many iVLEs employ static or repetitive audio effects that fail to account for the dynamic nature of feedback while performing a procedure. This inconsistency between the role of sensory input and its simulation fidelity reduces simulation realism, potentially limiting skill transfer and training effectiveness.

Pseudo-haptics and cross-modal perception

Pseudo-haptics leverages cross-modal perception, whereby information presented in one sensory modality alters perception in another [3,4]. Visual pseudo-haptics has been shown to convey properties such as stiffness, friction, mass, and texture by manipulating visual motion or deformation cues [11-13]. However, auditory cues are equally capable of influencing perceived force, hardness, and vibration, particularly through changes in frequency and loudness [14,15].

Research in pseudo-haptics and cross-modal interaction demonstrates that auditory cues can meaningfully alter tactile perception and motor behavior in virtual environments [3,14,15]. In this sense, auditory cues do not merely augment touch but participate in the construction of embodied procedural knowledge. A growing body of research suggests that audio-haptic coupling enhances motor performance in drilling tasks, even in low-fidelity simulations [14]. Yet, audio-based pseudo-haptics remains underexplored compared to its visual counterpart, representing an opportunity for innovation.

AI-generated audio and its role in simulation

Traditionally, the integration of realistic audio into immersive environments has been constrained by the labor-intensive nature of sound design. Creating dynamic, context-sensitive audio requires extensive manual curation and mixing, limiting scalability and consistency across training platforms [16,17].

Recent advances in generative artificial intelligence (AI) have transformed audio synthesis. Current models can generate realistic, temporally consistent audio from text [18], images [19], video [20], or audio inputs [21], and some systems integrate multiple modalities to enhance contextual sensitivity [22]. These developments suggest that AI-generated audio could dynamically respond to user actions in real time, such as changes in drilling depth or resistance.

Such developments extend earlier work on audio-based pseudo-haptics by introducing adaptive, context-aware sound generation that responds to interaction rather than functioning as static feedback [15,18-21]. From this perspective, AI-generated sound can be understood as an interactive sensory interface that mediates how learners perceive and regulate procedural action.

When applied to medical simulation, AI-generated audio offers several key advantages, including enhanced scalability by reducing reliance on expert sound designers, improved adaptability through real-time and context-aware feedback, and greater accessibility by lowering the costs associated with high-fidelity hardware.

Aside from simply providing environmental enhancement, AI-generated audio has the potential to function as a pseudo-haptic channel, reinforcing tactile perception in the absence of tactile cues.

Educational and clinical implications

The integration of AI-generated audio into iVLEs can have meaningful implications for medical education. First, it can provide a cost-effective alternative to traditional haptic devices, expanding access to psychomotor training in resource-constrained settings [2,23]. Second, it aligns with principles of embodied and situated cognition, which emphasize learning through multisensory interaction within realistic contexts [4,5]. Within medical education curricula, this approach is particularly relevant for early psychomotor training contexts such as pre-clinical skills laboratories or emergency medicine boot camps, where learners first encounter procedures that rely on subtle sensory cues. Novice trainees may benefit most, as auditory augmentation can help them attend to sensory features that experienced clinicians recognize implicitly. In this context, AI-generated audio can support formative learning and deliberate practice by reinforcing perceptual cues without replacing instructor guidance or high-fidelity simulation when available. From a theoretical perspective, this proposal aligns with principles of psychomotor learning, particularly sensory calibration and perceptual attunement, whereby learners refine motor output through multisensory feedback integration [24-26]. AI-generated auditory augmentation may potentially allow for the enhancement of perceptual-motor coupling during early skill acquisition. Third, it may enhance skill transfer by more closely approximating the sensory cues relied upon in real clinical environments.

It should be noted that audio-based pseudo-haptics is not a replacement for physical haptic devices where such technology is available. Instead, it represents a complementary modality that can enhance realism and learning outcomes when conventional haptics are impractical.

An example experimental approach

An example of this approach is exploring whether AI-generated audio can influence perceived tactile cues during simulated medical procedures in a meaningful manner. Our prior work has developed an iVLE that incorporates virtual reality (VR) to model an intraosseous (IO) drilling task [27], a procedure that relies heavily on auditory and tactile cues given limited visual feedback. During IO access, medical professionals depend on changes in resistance and corresponding changes in auditory pitch and intensity to confirm entry into the medullary cavity, emphasizing the importance of auditory-tactile integration for procedural accuracy and safety [9,10].

Within this proposed experiment, participants will perform a virtual drilling task under different auditory conditions, including real-world recordings of clinical drilling and AI-generated audio designed to dynamically reflect changes in material interaction. This design is informed by prior work showing that auditory feedback can influence motor performance and material perception in drilling-based tasks, even in low-fidelity or pseudo-haptic environments [14,15]. Rather than incorporating physical force-feedback devices, the simulation will leverage sound as a sensory substitute to reinforce tactile cue perception through cross-modal pseudo-haptics.

Perceived tactile cues, task realism, user experience, and engagement will be evaluated using standardized simulation assessment instruments, including the Michigan Standard Simulation Experience Scale (MiSSES) [28] and the NASA Task Load Index (NASA-TLX) [29]. Although results from this work are forthcoming, the aim of this experimental approach is to determine whether AI-generated audio may function as a pseudo-haptic channel, enhancing sensory realism and supporting psychomotor skill development in iVLEs without the need for specialized and costly haptic hardware.

Challenges and future directions

Despite its promise, several challenges must be addressed before AI-generated audio can be widely adopted in medical simulation. Although AI-generated audio can be advantageous with respect to scalability and accessibility, its use requires validation. Learners may place undue trust in simulated sensory cues if they assume the feedback fully reflects real-world tactile cues. To limit this risk, acoustic signatures representing procedural interactions should be validated by experienced clinicians and calibrated to reduce the potential for negative transfer to real-world procedures [30]. Furthermore, AI-generated audio should be treated as a complementary training modality that supports, but doesn’t replace, supervised hands-on practice and physical simulation [31].

Future research should examine how trainees integrate AI-generated auditory cues with visual and proprioceptive information, assess whether audio-based pseudo-haptics can improve psychomotor skill transfer to real-world practice, and explore the pedagogical as well as ethical implications of AI-mediated sensory feedback in healthcare training.

Conclusions

As iVLEs continue to evolve, audio should no longer be treated as a secondary aesthetic element. Many medical procedures rely on auditory cues to support the perception of tactile cues and decision-making, yet these are often ignored in iVLEs. AI-generated audio offers a novel and scalable alternative to enhance pseudo-haptics, particularly for psychomotor skills that rely on sound-tactile cue interactions. By treating sound as a functional element of tactile perception, medical education can move toward more accessible, highly immersive, realistic iVLEs that better reflect real-world clinical practice. From this viewpoint, simulation can be understood not only as the replication of physical reality but also as the design of interactive perceptual systems whereby sound, touch, and vision jointly scaffold embodied expertise.

The experiment proposed here highlights how this approach is beginning to be explored in practice. By applying AI-generated audio to a simulated IO drilling task, emerging research indicates a feasible approach for exploring audio-based pseudo-haptics within iVLEs. Although results are not yet available, such experiments demonstrate how audio can be intentionally designed to support psychomotor skill development in scenarios where traditional haptic technologies are impractical or inaccessible. Finally, although AI-generated pseudo-haptic audio offers scalability and accessibility benefits, its use must be carefully considered. Learners may place undue trust in AI-generated cues if they assume that the simulated feedback accurately reflects real-world tactile cues. To limit this risk, acoustic representations of tactile interactions should be validated by experienced clinicians and calibrated to reduce the potential for negative transfer. AI-generated audio should be used as a supplementary training tool that complements, rather than replaces, supervised hands-on practice and physical simulation. Its potential lies in increasing access to scalable iVLEs for medical education. Additional research and cross-disciplinary collaboration will help guide the effective use of auditory pseudo-haptics in medical training simulations.
